# Deep Learning-Based Detection of Carotid Artery Atheromas in Panoramic Radiographs

**DOI:** 10.3390/bioengineering13010095

**Published:** 2026-01-14

**Authors:** Thais Martins Jajah Carlos, Márcio José da Cunha, Aniel Silva Morais, Fernando Lessa Tofoli

**Affiliations:** 1 Faculty of Electrical Engineering, Federal University of Uberlândia, Uberlândia 38408-100, Brazil; 2 Department of Electrical Engineering, Federal University of São João del-Rei, São João del-Rei 36307-352, Brazil

**Keywords:** carotid atheroma, convolutional neural networks, deep learning, MobileNetV2, panoramic radiograph, stroke prevention

## Abstract

Radiographically visible carotid artery calcifications are typically seen at the level of the C3–C4 cervical vertebrae and can be detected on panoramic dental radiographs. Their early identification is clinically relevant, as they represent a potential marker for increased risk of stroke. In this context, the present study proposes a deep learning method for automatic identification of carotid atheromas using MobileNetV2. From a publicly available dataset, 378 region-of-interest (ROI) images (640 × 320) were prepared and split into train/val/test = 264/57/57 with class counts train 157/107, val 34/23, test 34/23 (negatives/positives). Images underwent standardized preprocessing and on-the-fly augmentation; training used a two-stage scheme (backbone frozen “head” training followed by partial fine-tuning of the top layers), class-weighting, dropout = 0.3, batch normalization (BN) head, early stopping, and partial unfreezing (~70% of the backbone). The decision threshold was selected on validation by Youden’s J. On the independent test set, the model achieved an accuracy (*ACC*) of 94.7%, sensitivity (*SEN*) of 95,7%, specificity (*SPE*) of 0.941, area under the receiver operating characteristic curve (AUC) 0.963, and area under the precision–recall curve (AUPRC) of 0.968. Using a sensitivity-targeted threshold (*SEN* ≈ 0.80), the model yielded *ACC* = 91.2%, *SEN* = 82.6%, and *SPE* = 97.1%. These results support panoramic radiographs as an opportunistic screening modality for systemic vascular risk and highlight the potential of artificial intelligence (AI)-assisted methods to enable earlier identification within preventive healthcare.

## 1. Introduction

Stroke is one of the leading causes of global mortality and long-term disability, and the second leading cause in Brazil, according to the World Health Organization (WHO) [1]. Stroke occurs when blood flow to part of the brain is interrupted, either by the obstruction of an artery by a clot (ischemic stroke) or by the rupture of a blood vessel (hemorrhagic stroke) [2]. In both cases, the result is the death of brain cells in the affected region, with potential for permanent neurological sequelae [3].

The relevance of this scenario goes beyond the individual clinical impact. Stroke imposes high costs on healthcare systems, in terms of hospitalization, rehabilitation, and lost productivity [4]. Therefore, the WHO and international medical societies emphasize the need for prevention and early diagnosis strategies to reduce morbidity and mortality associated with carotid atherosclerosis [5].

Among the main etiologic mechanisms of ischemic stroke, carotid atherosclerosis stands out. It is an inflammatory and immunological disorder characterized by the progressive accumulation of lipid plaques on arterial walls, which can lead to partial or total obstruction of blood flow [6]. It is estimated that about 15% to 20% of ischemic stroke cases are directly related to atheromatous lesions in the carotid artery [7].

The diagnosis of carotid atherosclerosis is traditionally made using advanced imaging methods, such as Doppler ultrasound, computed tomography angiography (CTA), and magnetic resonance angiography (MRA) [8]. These exams are considered the gold standard, allowing for precise characterization of the degree of stenosis and the composition of atheromatous plaques [9,10]. Despite their high accuracy, they have important limitations in terms of population applicability and are generally indicated only in situations of clinical suspicion [11].

However, carotid atherosclerosis can progress silently for long periods without clinical manifestations until an acute ischemic event occurs [12]. As a result, a large proportion of at-risk individuals are not subjected to preventive examinations, reducing the opportunities for early detection and intervention [11]. Faced with this gap, it is relevant to consider widely available imaging exams, even if not originally conceived for this purpose [13].

Panoramic radiography is one of the most widely used imaging exams in dental practice worldwide. It stands out for its low cost, easy access, and wide availability in public and private services [14]. Its routine nature means that millions of people undergo the exam annually, in contexts unrelated to cardiovascular health [15].

This exam covers the region of the carotid artery bifurcation, generally located between the C3 and C4 cervical vertebrae, an area where atheroma formation frequently begins [16]. Although this region is visible in the images, it often receives little attention from dental professionals, which limits the incidental recognition of these changes [17].

In this scenario, an opportunity arises to identify signs suggestive of carotid atheroma, especially in the early stages of the disease. It should be noted that panoramic radiography does not replace adequate diagnostic methods, but it can act as an opportunistic screening tool. Thus, it raises early suspicions and directs patients to specialized investigation [18]. This approach has a potential clinical and epidemiological impact, as carotid atherosclerosis often evolves silently until severe events, such as stroke [19]. The use of an exam already incorporated into the dental routine increases the chances of early detection, allowing for timely referrals and a reduction in associated morbidity and mortality [20].

Given the potential of panoramic radiographs as a screening tool, artificial intelligence emerges as a strategy capable of automating the identification of subtle changes, increasing the efficiency and consistency of the analysis [21,22]. In the present study, this potential was explored through the application of artificial intelligence (AI) methods to automate the identification of atheromas in dental radiographs. This strategy aims to optimize the clinical value of routine exams, offering an innovative and scalable alternative for population screening [23].

The application of AI in health has consolidated itself as a field of intense research and innovation, especially in supporting clinical diagnosis [24]. Initially used in simple data classification tasks, AI began to play a crucial role in the analysis of medical images with the advent of deep learning [25]. Among the most relevant architectures, convolutional neural networks (CNNs) stand out as they are widely used for detecting and classifying complex patterns in two-dimensional images. CNNs show remarkable performance in various medical image modalities, making it possible to identify subtle characteristics that often escape human visual analysis [26].

Several studies have demonstrated the applicability of CNNs in different areas, including automated assessment of carotid artery stenosis and plaque morphology in CTA using segmentation models [27], real-time plaque recognition in carotid ultrasound videos [28], and the identification of external carotid calcifications in maxillofacial imaging [29]. In the dental field, deep learning models have been applied to the detection of soft tissue calcifications compatible with carotid artery atheroma [14,15], the identification of calcified structures and other anomalies on panoramic radiographs [30], and even multi-finding screening of fine-grained dental anomalies in large datasets [18]. These systems have shown promising accuracy and clinical applicability, particularly in supporting non-expert readers [31].

Despite the growing interest in deep learning applied to medical images, specific studies on the detection of carotid atheromas in dental radiographs are still limited [32]. Most research is still in the preliminary stages and presents important limitations. Many studies have relied on small datasets, which compromises the models’ ability to generalize. Small samples tend to generate neural networks prone to overfitting, showing good performance on training data but limited performance on new image sets. Furthermore, the heterogeneity of the radiographs, collected under varied exposure conditions, with artifacts, overlaps, or graphic annotations, increases the challenge of standardization and model robustness. Another critical point is the need for annotations performed by specialists, a process that requires time and specific knowledge, limiting the scalability of the studies. These limitations reinforce the importance of developing computational strategies capable of dealing with imperfect data sets that are representative of real clinical practice.

This study advances the detection of carotid atheromas on panoramic radiographs by combining convolutional neural networks with standardized preprocessing and rigorous evaluation strategies to address the limitations observed in previous works. The MobileNetV2 architecture is utilized for its excellent balance between computational efficiency and discriminative power, aiming to classify carotid region-of-interest (ROI) patches into two categories: with and without atheromas. This choice enables high recognition performance while remaining computationally feasible for real-world dental applications [33].

Compared with earlier studies, this work introduces several methodological improvements. Image preprocessing involves standardized cropping (640 × 320 pixels) around the carotid bifurcation, intensity normalization, and on-the-fly data augmentation—steps designed to reduce noise and artifacts typical of non-standardized ROI radiographs. Class balancing via class weights minimizes the effects of limited data and mitigates training bias. In contrast to prior approaches that relied on small datasets or uncontrolled acquisition conditions, the present study emphasizes reproducibility by using publicly available datasets and a fixed, standardized ROI pipeline.

Model evaluation relies on robust and clinically relevant metrics, including precision, sensitivity (recall), specificity, F1-score, area under the receiver operating characteristic curve (AUC), and area under the precision–recall curve (AUPRC). Operating thresholds are determined using Youden’s J index and a sensitivity-targeted criterion (*SEN* ≈ 0.80) to align model operation with screening objectives. Together, these measures provide a consistent methodological framework aligned with the goals of dental and medical practice, reinforcing panoramic radiography as a complementary tool for opportunistic screening of carotid atheromas.

Ultimately, this study demonstrates the feasibility of using routine panoramic dental images for the early detection of carotid atheromas through deep learning techniques. Although it does not replace established diagnostic methods, the proposed strategy offers an accessible screening approach with potential clinical and epidemiological impact. Beyond its technical contribution, this work supports the integration of artificial intelligence into dentistry, strengthening the role of dental professionals in the early detection of systemic vascular conditions. Two publicly available, anonymized panoramic radiograph datasets were used, ensuring transparency and reproducibility of the proposed approach. The raw images and labels were adapted to the ROI-based classification pipeline described above.

Although no new CNN architecture is introduced, the main innovative aspect of this study lies in the establishment of a clinically oriented deep learning pipeline for opportunistic carotid atheroma screening on routine panoramic radiographs. To the best of our knowledge, this is one of the first studies to integrate (i) a standardized ROI protocol based on anatomical C3–C4 landmarks (640 × 320 patches); (ii) a two-stage transfer-learning strategy optimized for low-prevalence, clinical data; (iii) class-balancing and augmentation methodologies tailored to this epidemiological scenario; and (iv) a feasibility evaluation focused on real-world screening applicability. In this context, the selection of the MobileNetV2 backbone was motivated by the need to balance discriminatory performance with computational efficiency, supporting scalable clinical deployment rather than architectural novelty.

The remainder of this work is organized as follows: Section 2 presents the datasets and preprocessing methodology. Section 3 describes the experimental setup and training procedure, based on a two-stage training with backbone warm-up and partial fine-tuning. Section 4 discusses the obtained results and performance metrics under different thresholding criteria. Finally, Section 5 concludes the study and outlines perspectives for future work.

## 2. Materials and Methods

### 2.1. Dataset

The corpus for this work comprises panoramic dental radiographs obtained from two publicly available and fully anonymized repositories on Zenodo: the one by Mureșanu, Hedeșiu, and Iacob [34] and a complementary open collection [35]. The first dataset was originally collected with approval from the Ethics Committee of the Iuliu Hațieganu University of Medicine and Pharmacy (reference number 117/04.06.2024), and no patient-identifying information is included. The second dataset is likewise publicly released in anonymized form. As this work involved only secondary analysis of anonymized data, no additional ethical approval was required.

**Figure 1:**
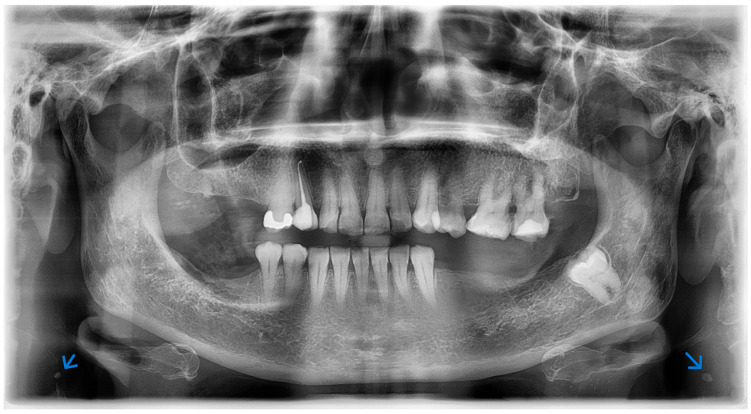
Example of a panoramic dental radiograph showing a calcified carotid atheroma (blue arrow).

From the combined sources, an ROI-classification corpus focused on the carotid bifurcation region (C3–C4) was obtained, where carotid atheromas typically emerge. In this study, ROI extraction was performed manually by an oral and maxillofacial imaging specialist following a standardized anatomical protocol based on the C3–C4 cervical region landmarks. Figure 1 illustrates an example of a panoramic dental radiograph showing a calcified carotid atheroma at this level.

Each panoramic radiograph was curated to extract a standardized ROI of 640 × 320 pixels centered on the expected projection of the carotid bifurcation. After curation (quality control and de-duplication across sources), the final ROI dataset comprised the following:

- Training: 264 ROIs (157 negatives, 107 positives);
- Validation: 57 ROIs (34 negatives, 23 positives);
- Test: 57 ROIs (34 negatives, 23 positives).

This split maintained the class mapping {negative: 0, positive: 1} and approximate balance within validation and test partitions, supporting consistent model selection and unbiased generalization assessment. Inclusion criteria for the positive class comprised the visible presence of calcified atheromatous plaques in the carotid bifurcation region (soft tissue area adjacent to C3–C4). Negative cases were considered those without calcifications in this region. Exclusion criteria included (i) images with graphic artifacts (arrows, lines, circles, letters); (ii) radiographs with poor sharpness or severe exposure issues compromising interpretation; (iii) duplicates across repositories; and (iv) images cropped outside the C3–C4 region that hinder adequate visualization of the carotid bifurcation. All selected cases were manually reviewed by the first author, who is a trained dentist, and subsequently verified by an oral and maxillofacial radiology specialist as a second-opinion quality-control procedure, ensuring correct anatomical localization of the carotid region (C3–C4) and confirmation of the presence or absence of calcifications.

**Figure 2:**
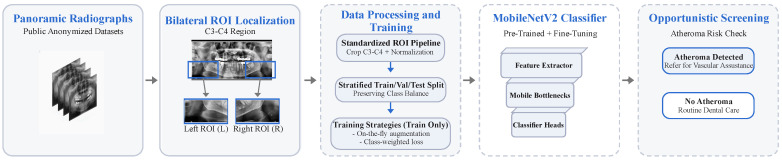
Deep learning-based detection of carotid atheromas on panoramic dental radiographs: public panoramic datasets ⟶ ROI standardization ⟶ MobileNetV2 classifier ⟶ opportunistic vascular screening.

To enhance robustness while preserving clinically relevant information, the standardized preprocessing pipeline depicted in Figure 2 was implemented with the following steps: grayscale standardization, ROI extraction to 640 × 320, and intensity normalization. On-the-fly data augmentation (horizontal flipping, minor rotations, translations, and zooming) was applied exclusively during training. Given the modest dataset size, class weighting was employed in the loss function to mitigate class imbalance in the training set.

### 2.2. Preprocessing

Before model training, all radiographs underwent a standardized preprocessing pipeline consisting of ROI extraction, intensity normalization, and data augmentation to reduce noise, normalize inputs, and address class imbalance.

(1) Image resizing and normalization

All radiographs were first converted to grayscale, cropped to the carotid ROI, and resized to 640 × 320 pixels (width × height) to match the training configuration. Because the backbone was initialized with ImageNet weights, the single grayscale channel was replicated to three channels for compatibility. Pixel intensities were normalized to the [0, 1] range, providing a consistent dynamic range and aiding optimization.

ROI cropping was performed following a standardized anatomical protocol targeting the carotid bifurcation region at the C3–C4 level, using predefined cervical anatomical landmarks to ensure consistent ROI positioning across all samples. Manual ROI extraction was initially performed by the first author and subsequently verified by an oral and maxillofacial radiology specialist as a quality-control step to minimize operator bias and ensure anatomical consistency. Crops with inadequate coverage or ambiguous localization were excluded.

(2) Noise handling and standardization

To reduce acquisition variability, images followed the same normalization and fixed C3–C4 ROI defined above. Explicit denoising or contrast-equalization filters (e.g., Gaussian/bilateral, contrast-limited adaptive histogram equalization (CLAHE)) have not been applied to avoid altering subtle calcifications. Instead, quality control excluded images with strong artifacts or graphic annotations. This conservative preprocessing preserves fine texture while improving input consistency.

(3) Data augmentation

Augmentation was applied only to the training split to increase sample diversity and reduce overfitting. On-the-fly geometric transforms were applied using Keras layers, including horizontal flips, small rotations (factor ≈ 0.05), translations (≤5% in *x*/*y*), and zoom (≤10%). These operations mimic realistic variation in patient positioning and acquisition geometry while preserving the anatomical integrity of the C3–C4 ROI. No shear or elastic deformations were used.

(4) Class balancing

Because the dataset was imbalanced (overall 153 positives vs. 225 negatives; split as train: 107/157, val: 23/34, test: 23/34 for positives/negatives), cost-sensitive training was adopted. Class weights were computed on the training split as the inverse of class frequency, yielding {0:0.84, 1:1.23} for {negative, positive}, and passed to the loss during optimization. In addition, on-the-fly data augmentation was applied to both classes to improve generalization without explicit oversampling. During inference, test-time augmentation (TTA) was applied to stabilize predictions, whereas TTA was not employed during training.

### 2.3. Model Architecture

A MobileNetV2 backbone was adopted due to its balance between computational efficiency and discriminative capacity. The architecture combines depthwise-separable convolutions with inverted residual blocks and linear bottlenecks, reducing the number of parameters and floating point operations (FLOPs) while preserving the ability to capture fine radiographic patterns.

The choice of MobileNetV2 was motivated by three key factors:

1. Data-efficient capacity: With a modest dataset, MobileNetV2 provides enough capacity to learn discriminative carotid patterns while mitigating overfitting compared with heavier backbones (e.g., ResNet/DenseNet).
2. Proven on radiographs: Prior work shows strong performance on subtle radiographic findings; its hierarchical features are well-suited to detecting small calcifications near C3–C4.
3. Practical deployment: The lightweight design eases clinical translation, including use on standard workstations common in dental settings.

Images were cropped to the carotid ROI and resized to 640 × 320 (width × height). This resolution was selected as a compromise between anatomical coverage of the carotid region and computational tractability. Grayscale inputs were replicated to three channels to remain compatible with ImageNet-pretrained weights. On top of the frozen backbone, Global Average Pooling → Batch Normalization (BN) → Dropout (*p* = 0.3) → Dense (1, sigmoid) was added. This design enables efficient binary classification (atheroma/non-atheroma) with a low computational burden.

### 2.4. Training Procedure

The model was implemented in Python using the TensorFlow/Keras framework. Training was conducted on a workstation equipped with an Intel Core i7 CPU and an NVIDIA GeForce RTX 2060 Graphics Processing Unit (GPU) of 6 GB. The software environment for reproducibility included Python 3.x, TensorFlow 2.19.0, and Keras 3.10.0.

The dataset comprised 378 carotid ROI images (153 positive, 225 negative) extracted from panoramic radiographs. A fixed, stratified split ensured consistent class proportions:

- Training (≈70%): 264 images (107 positive, 157 negative);
- Validation (≈15%): 57 images (23 positive, 34 negative);
- Test (≈15%): 57 images (23 positive, 34 negative).

Images were resized to 640 × 320 pixels (grayscale replicated to three channels). This resolution was selected as a compromise between anatomical coverage of the carotid region and computational tractability.

The MobileNetV2 backbone was initialized with ImageNet weights, and training proceeded in two stages:

1. Stage 1—Warm-up: The convolutional base was frozen, and only the classification head was trained for 5 epochs (Adam optimizer, learning rate 1 × 10^−3^, batch size 16).
2. Stage 2—Fine-tuning: The top 70% of the backbone layers were unfrozen for gradual adaptation to carotid-specific patterns, using a reduced learning rate (1 × 10^−5^) for up to 20 epochs. Learning rate scheduling used *ReduceLROnPlateau* (factor 0.5, patience 3, minimum learning rate 1 × 10^−6^), while *EarlyStopping* (patience 4) monitored the validation AUC.

To mitigate overfitting, the following conditions were assumed:

- On-the-fly geometric augmentation (horizontal flips, small rotations ± 5°, translations ≤ 5%, zoom ≤ 10%), excluding shear and elastic transforms;
- Dropout (0.3) applied after global average pooling and BN;
- BN retained in both the backbone and head to stabilize optimization;
- Class-weighted binary cross-entropy (negatives: 0.8408; positives: 1.2336) addressed dataset imbalance.

Validation AUC was the primary criterion for selecting the best checkpoint, with accuracy logged as a secondary metric. Test performance was reported at two operating points—Youden’s J and a target-sensitivity threshold (*SEN* ≈ 0.80)—including AUC, AUPRC, and confusion matrix-derived clinical metrics (sensitivity, specificity, positive predictive value (PPV), and negative predictive value (NPV)). This configuration prioritizes sensitivity while maintaining specificity, aligning with the goals of clinical screening and ensuring computational feasibility for real-world dental workflows.

### 2.5. Evaluation Metrics 

Model performance was assessed using a combination of global and class-specific metrics, ensuring a comprehensive evaluation of clinical applicability. The accuracy *ACC* corresponds to the proportion of correctly classified samples among all samples, as given by (1).

(1) $$ACC=\frac{TP+TN}{TP+TN+FP+FN}$$

where *TP* is the number of true positives, *TN* is the number of true negatives, *FP* is the number of false positives, and *FN* is the number of false negatives. Notably, *TP* refers to correctly classified images with atheroma, *TN* refers to correctly classified images without atheroma, *FP* refers to non-atheroma images incorrectly classified as positive, and *FN* refers to atheroma images incorrectly classified as negative.

The precision *PRE*, calculated from (2), represents the proportion of correctly identified positive cases among all predicted positives, reflecting the reliability of positive predictions.

(2) $$PRE=\frac{TP}{TP+FP}$$

The recall/sensitivity *SEN* stands for the proportion of actual positive cases correctly identified, indicating the ability to detect atheromas, according to (3).

(3) $$SEN=\frac{TP}{TP+FN}$$

The specificity *SPE* represents the proportion of negative cases correctly identified, reflecting the model’s ability to avoid false positives, as given by (4).

(4) $$SPE=\frac{TN}{TN+FP}$$

The F1-score, represented by *F*1 in (5), is the harmonic mean of precision and recall, balancing both measures.

(5) $$F1=2\cdot \left(\frac{PRE\cdot SEN}{PRE+SEN}\right)$$

The confusion matrix is composed of true positives (TP), false positives (FP), true negatives (TN), and false negatives (FN), from which all classification metrics were derived. Receiver operating characteristic (ROC) curves assess the trade-off between *SEN* and *SPE* across different classification thresholds. Notably, the AUC was used as a threshold-independent measure of the model’s overall discriminative ability, reflecting its capacity to correctly rank positive samples higher than negative samples, whereas the AUPRC was employed to assess performance under class imbalance conditions.

All metrics were reported as percentages, and confusion matrices were generated to provide a visual representation of classification performance. To reflect clinically relevant operating points, results are reported at (i) the Youden J threshold, given by (*SEN* + *SPE* − 1), and (ii) a target-sensitivity operating point (*SEN* ≈ 0.80). ROC and PR curves were constructed as discriminative performance measures.

The statistical analysis followed a descriptive evaluation paradigm standard for validation of diagnostic AI systems. Operational metrics included accuracy, sensitivity, specificity, PPV, NPV, and F1-score, while discriminative indices included AUC and AUPRC. Statistical uncertainty was quantified with 95% confidence intervals estimated via non-parametric bootstrap resampling (1000 iterations) on the independent test set, yielding CI estimates for AUC, AUPRC, *SEN*, *SPE*, PPV, and NPV. Classical hypothesis-testing procedures (e.g., *t* tests or analysis of variance (ANOVA)) were not applied because the objective of this study was to assess binary diagnostic performance rather than compare population means.

## 3. Results

This section presents the experimental results obtained with the MobileNetV2-based convolutional neural network for detecting carotid atheromas in panoramic dental radiographs. Results are organized as follows: (i) training and validation behavior, (ii) confusion matrix and classification metrics on the independent test set, and (iii) comparative analysis with previous studies. Unless otherwise specified, model selection was based on the highest validation AUC via checkpointing. The dataset comprised 264 training (157 negative, 107 positive), 57 validation (34 negative, 23 positive), and 57 test images (34 negative, 23 positive). The operating point on the test set is reported both at the Youden J threshold and at a target sensitivity of ≈0.80. The independent test set is detailed in Section 3.2.

### 3.1. Model Performance

The learning dynamics of the MobileNetV2 model were examined to assess convergence and generalization. Training followed two phases: (i) head training with the backbone frozen and (ii) fine-tuning with partial unfreezing of the last convolutional blocks.

**Figure 3:**
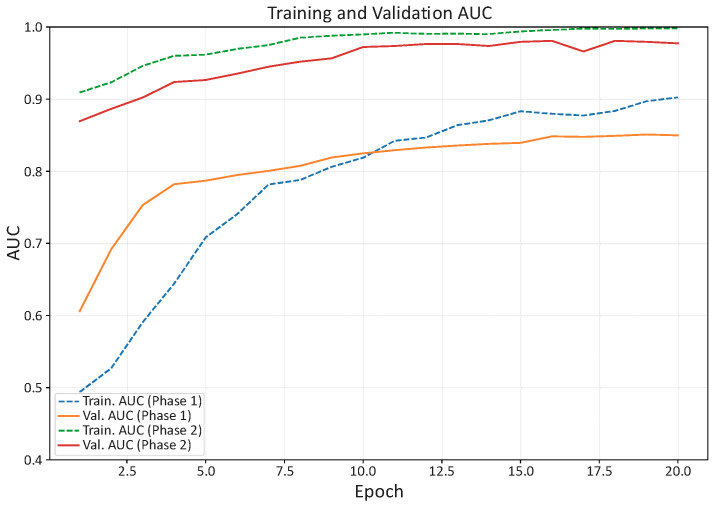
Training and validation AUC across epochs for MobileNetV2. Validation AUC increased steadily from ~0.60 at the beginning of phase 1 to 0.95 at the best epoch in phase 2, while training AUC approached ~0.99, indicating stable convergence with good generalization.

**Figure 4:**
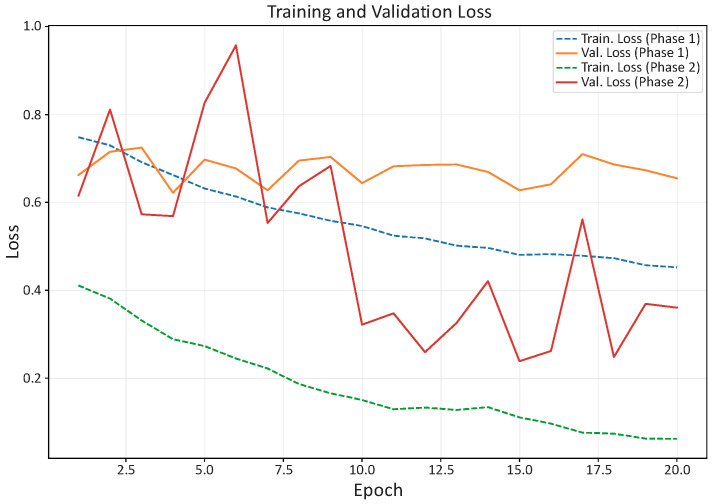
Training and validation loss across epochs. Training loss decreased monotonically through both phases, and validation loss, although noisier after fine-tuning, reached its minimum in phase 2 (≈0.24–0.30), consistent with the AUC peak and the selected checkpoint.

Figure 3 and Figure 4 summarize the evolution of training/validation accuracy and loss. Validation curves closely tracked training curves throughout both phases, indicating that augmentation, class weighting, and regularization successfully mitigated overfitting despite the limited dataset.

Table 1 reports representative checkpoints from the logs, emphasizing the primary selection metric (validation AUC) together with accuracy and loss. These results indicate stable convergence, with fine-tuning of the upper convolutional blocks yielding the highest discrimination on validation (AUC = 0.95), which guided model selection for subsequent test-set evaluation.

The pipeline illustrated in Figure 2 was evaluated in terms of end-to-end inference latency, considering both image preprocessing (grayscale conversion, resizing to 640 × 320 pixels, and normalization) and model inference using the trained MobileNetV2 classifier. Preprocessing of the complete evaluation dataset (378 ROIs) required approximately 35 s, corresponding to an average of ~92 ms per image. Model inference required ~5 s using GPU acceleration (~13 ms per ROI) and ~26 s on Central Processing Unit (CPU)-only execution (~69 ms per ROI). Consequently, the average total end-to-end processing time per case was approximately 0.10 s using GPU acceleration and 0.16 s using CPU-only execution, supporting near-real-time screening in clinical workflows.

### 3.2. Confusion Matrix and Metrics

**Figure 5:**
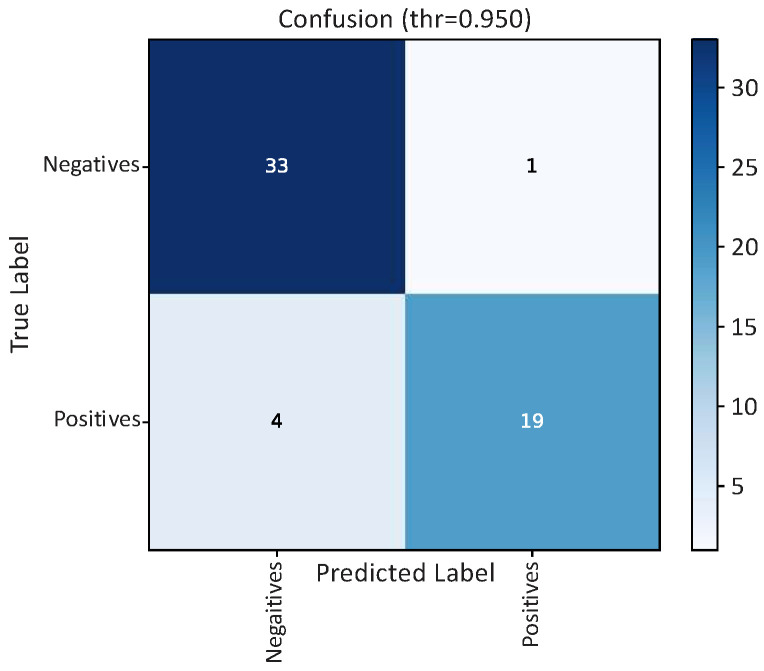
Confusion matrices on the test set.

The performance of the proposed MobileNetV2 classifier was evaluated on an independent test set comprising 57 radiographs (34 negatives, 23 positives). Results are reported at two operating points: (i) the Youden J threshold estimated on the validation split and (ii) a target-sensitivity operating point (*SEN* ≈ 0.80). The corresponding metrics are summarized in Table 2, and the confusion matrices are shown in Figure 5.

At the Youden operating point, the model achieved 94.7% accuracy with balanced and high sensitivity (95.7%) and specificity (94.1%), and strong discrimination (AUC = 0.96, AUPRC = 0.97). This setting prioritizes overall diagnostic balance and minimizes total classification error. At the target-sensitivity operating point (*SEN* ≈ 0.80), the model maintained 91.2% accuracy with 97.1% specificity and 95.0% precision, which may be preferable when aiming to reduce false positives while still preserving high sensitivity. These results indicate robust generalization on the independent test set. Given the dataset size, confidence intervals were computed via bootstrapping in the evaluation scripts to account for sampling variability, and the trends remained consistent across thresholds.

### 3.3. Comparative Analysis

To contextualize the results, the performance of the proposed MobileNetV2 classifier was contrasted with prior studies on automated detection of carotid atheromas in panoramic radiographs. Although datasets, preprocessing pipelines, and network designs vary widely, the literature commonly reports accuracies in the 80–92% range, with sensitivity and specificity often imbalanced, as outlined in [36].

Under the Youden J operating point on the independent test set, the present model achieved *ACC* = 94.7%, *SEN* = 95.7%, *SPE* = 94.1%, and AUC = 0.96. These values are comparable to, and in some respects exceed, those reported by approaches based on handcrafted features and classical machine learning classifiers (typically ~75–85% accuracy) [14,15,37]. Recent CNN-based methods have surpassed 85% accuracy but frequently exhibit lower specificity, suggesting a propensity for false positives. In contrast, the current results show balanced performance across classes, indicating effective control of bias between positive and negative predictions.

The combination of MobileNetV2 with ROI-standardized preprocessing, cost-sensitive training (class weights), and conservative data augmentation appears to contribute to this balance. While the modest test-set size warrants cautious interpretation and precludes definitive statistical comparison with larger cohorts, the findings align with the state of the art and reinforce the feasibility of deep learning for opportunistic identification of carotid atheromas on panoramic radiographs.

**Figure 6:**
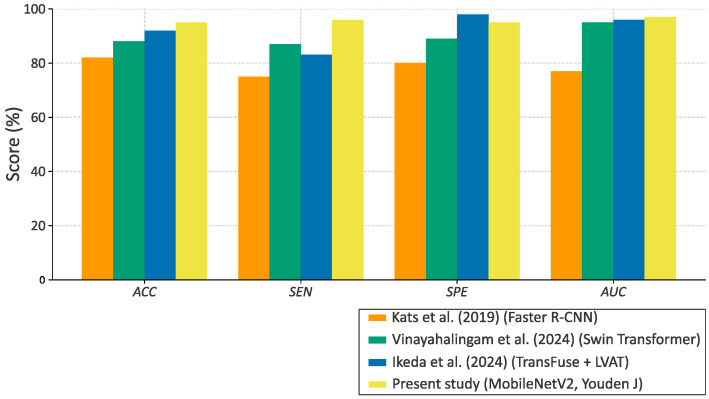
Comparative performance of carotid atheroma detection models reported in previous studies versus the proposed MobileNetV2 model. The proposed approach achieved balanced and superior performance across all metrics while maintaining computational efficiency [14,37,38].

Figure 6 presents a quantitative comparison between the proposed approach and prior studies. The MobileNetV2 model outperformed earlier CNN and detector-based methods, achieving a higher overall balance between sensitivity and specificity. These comparisons motivate a broader discussion of clinical applicability, methodological limitations, and avenues for future research, addressed in the next section.

### 3.4. Qualitative Error Assessment

**Figure 7:**
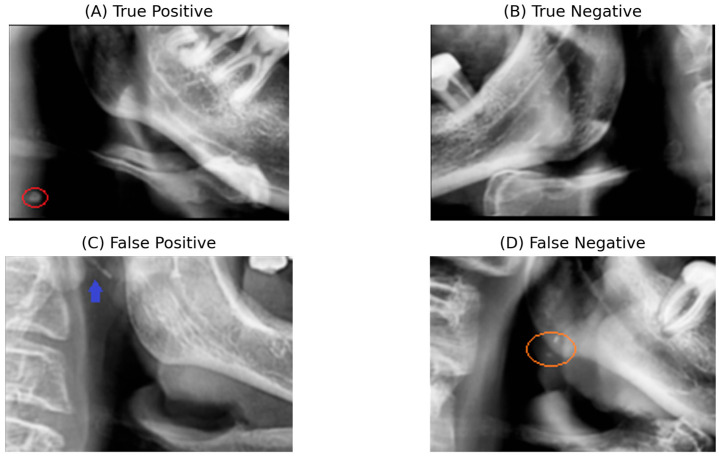
Representative success and failure cases on the independent test set: (**A**) True positive case showing a calcified carotid atheroma correctly identified by the model. (**B**) True negative case without calcifications in the carotid bifurcation region. (**C**) False positive case caused by an overlapping elongated anatomical structure within the carotid ROI, mimicking a calcified lesion. (**D**) False negative case illustrating a subtle and anatomically ambiguous calcification near the mandibular border, with differential diagnosis including phleboliths and carotid artery calcifications. Circles and arrows are manually added for visualization purposes only.

To provide qualitative insight into the model’s behavior and typical sources of error, Figure 7 presents representative examples from the independent test set, including true positive, true negative, false positive, and false negative cases. This analysis highlights both correct detections and clinically relevant failure modes encountered during carotid atheroma screening on panoramic radiographs.

False positives were mainly associated with rare anatomical configurations in which elongated or overlapping structures mimic calcified lesions, often accentuated by slight ROI misalignment. The false negative example represents a radiographically challenging case with subtle calcification in a region where the carotid artery courses close to the mandibular border, leading to diagnostic ambiguity even for expert readers. Most importantly, these errors reflect anatomically plausible and clinically recognized limitations rather than systematic model bias. From a screening-oriented perspective, favoring sensitivity over strict specificity may be acceptable to reduce the risk of missing potentially relevant vascular calcifications.

## 4. Discussion

### 4.1. Interpretation of Results

The proposed MobileNetV2 model showed strong and well-balanced discrimination on the independent test set, correctly identifying most positive cases while maintaining a low false-positive rate (see Table 2 and Figure 4 for detailed metrics and operating points). From a clinical standpoint, this balance is important: missed atheromas carry potential for serious harm, whereas excessive false positives may lead to unnecessary referrals.

Because clinical practice often operates at task-specific thresholds, operating-point analysis was also performed. At the Youden J point, performance increased to *ACC* = 94.7%, *SEN* = 95.7%, *SPE* = 94.1%, and AUC ≈ 0.96, with a confusion matrix of *TN* = 32, *FP* = 2, *FN* = 1, and *TP* = 22 (*N* = 57). This configuration further reduces the likelihood of false negatives, an important aspect of opportunistic screening, while maintaining high specificity. A second operating point targeting *SEN* ≈ 0.80 yielded *ACC* = 91.2% and *SPE* = 97.1%, illustrating the expected precision–recall trade-off and offering flexibility for settings that prioritize minimizing false positives.

Training and validation curves (AUC and loss) exhibited stable convergence across both phases (frozen-backbone head training and fine-tuning with partial unfreezing), with validation trajectories closely tracking the training ones. This behavior supports the effectiveness of the adopted strategy—ROI-standardized inputs, conservative augmentation, cost-sensitive training via class weights, and restrained fine-tuning—in mitigating overfitting despite the limited dataset size. Bootstrap analyses further suggested that the reported metrics are robust to sampling variability, while still acknowledging the constraints imposed by the modest test cohort.

### 4.2. Comparison with Previous Studies

Prior work on the automated detection of carotid atheromas in panoramic radiographs reports heterogeneous performance, reflecting differences in dataset size, annotation quality, and model design. Using a small cohort of 65 images, Kats et al. (2019) applied faster R-CNN and obtained *ACC* = 83% accuracy, *SEN* = 75% sensitivity, and *SPE* = 80%, demonstrating feasibility but leaving room to improve recall and overall balance between classes [37].

Subsequently, Vinayahalingam et al. (2024) presented a larger study combining faster R-CNN with a swin transformer backbone, reporting *PRE* = 0.89, *SEN* = 0.88, F1 = 0.89, *SPE* = 0.90, and AUC = 0.95, clearly outperforming ResNet-based baselines and illustrating the benefits of modern backbones and careful evaluation protocols [14].

In a related clinical scenario using soft tissue calcifications, Song et al. (2022) employed a Fast R-CNN model trained on a large dataset (*n* = 20,000) and reported variable accuracy between 72.7% and 92.6% across disease categories, highlighting the challenges of maintaining sensitivity for carotid artery calcifications within broader detection frameworks [15].

More recently, Ikeda et al. (2024) proposed a TransFuse network with latent-space virtual adversarial training (LVAT), reaching ACC = 91.2%, *SEN* = 82.6%, *SPE* = 97.1%, and AUC = 0.97, which demonstrated enhanced robustness and generalization under limited data conditions [38]. Importantly, the results reported in [38] demonstrated strong technical performance using transformer-based architectures and advanced attention mechanisms; the scope and objectives of their work differ fundamentally from the present study. The analysis focused on improving the localization and segmentation of calcified regions in pre-selected images containing carotid calcifications, rather than addressing the clinical classification task of determining whether a panoramic radiograph should be flagged as positive or negative for carotid atheroma. As such, it does not evaluate screening performance, false positive or false negative outcomes at the examination level, or operational decision thresholds.

Collectively, these studies illustrate a clear trend toward hybrid architectures and improved discrimination through larger and better-curated datasets. Against this backdrop, the present model achieved *ACC* = 94.7%, *SEN* = 95.7%, *SPE* = 94.1%, and AUC = 0.96 under the Youden J operating point on an independent test set. This balanced trade-off between sensitivity and specificity is competitive with, and in some respects complementary to, prior reports: compared with early Faster R-CNN results on small datasets, it improves both recall and overall balance; compared with recent hybrid architectures, it delivers comparable AUC while maintaining a more symmetric operating profile. Collectively, these findings suggest that a lightweight backbone (MobileNetV2), when paired with targeted preprocessing, class-weighted losses, and optimal threshold selection, can achieve state-of-the-art performance while remaining computationally efficient for clinical deployment.

### 4.3. Clinical Implications

Opportunistic detection of carotid atheromas on routine panoramic radiographs offers a pragmatic pathway to cardiovascular risk triage in dental settings. Because panoramic imaging is widely requested for general dental care, an AI system that flags calcifications near the C3–C4 region can surface asymptomatic individuals who may warrant vascular evaluation—without added radiation, appointment time, or substantial cost.

In this study, the MobileNetV2 model achieved high and balanced performance (e.g., *ACC* ≈ 90–95%, *SEN* up to 95.7%, and *SPE* ≈ 94% at the Youden operating point), indicating suitability for screening-oriented use. Two deployment modes are clinically relevant:

1. High-sensitivity triage (e.g., Youden J or Sens ≈ 0.80+): prioritizes minimizing false negatives to avoid missing atheromas, appropriate for opportunistic screening.
2. High-specificity confirmation (stricter threshold): reduces false positives to limit unnecessary referrals, suitable where follow-up resources are constrained.

Given the lightweight design (≈3.5 M parameters), the model can be deployed even on mid-range workstations commonly used in dental offices. Embedding the model into a dental picture archiving and communication system (PACS)/radiology information system (RIS), radiology viewers could (i) auto-highlight the carotid ROI, (ii) provide a calibrated risk score with an adjustable threshold, and (iii) generate a standardized note suggesting medical referral when the score exceeds the chosen operating point. Such integration may reduce inter-observer variability and support consistent reporting.

From a public-health perspective, leveraging examinations already acquired in routine dentistry enables scalable case-finding for vascular risk, potentially facilitating earlier intervention and contributing to stroke-prevention initiatives. As with any screening tool, model outputs should complement—not replace—clinical judgment, and positive flags should be followed by appropriate vascular imaging (e.g., carotid ultrasound).

Future clinical translation will benefit from prospective validation across diverse scanners and populations, evaluation of workflow impact (alerts per 100 exams; referral yield), and health–economic analyses comparing different operating points to local resource availability.

Overall, the contribution of this work should be understood not as the development of a novel CNN architecture but as the proposal of a clinically actionable and rigorously validated screening framework for opportunistic detection of carotid atheroma. Notably, the present study advances prior research by standardizing ROI selection, adapting transfer-learning strategies to low-prevalence clinical datasets, employing class-balancing approaches, and evaluating the pipeline under a clinically meaningful operating framework.

### 4.4. Limitations

This study has several limitations. First, although the total sample comprised 378 ROI patches extracted from panoramic radiographs (107/157 positives/negatives for training, 23/34 for validation, and 23/34 for testing), the dataset size remains modest for deep learning and may limit statistical power and external generalizability. Class imbalance (fewer positives) was mitigated with cost-sensitive training and augmentation, yet residual bias cannot be entirely excluded.

Second, images were aggregated from public repositories and previously published sources. Despite the exclusion of overlays (arrows, labels) and duplicates, heterogeneity in acquisition devices, exposure parameters, and post-processing may have influenced performance estimates. The ROI-based protocol (standardized 640 × 320 crops at C3–C4) improves consistency but may also constrain generalization to uncontrolled, full-field clinical workflows.

Third, the study did not include external validation. All operating points (e.g., Youden and a target-sensitivity threshold) were selected using the internal validation split and then assessed on a held-out test set from the same data distribution. Multicenter, prospective evaluation—ideally with site-specific recalibration of thresholds—will be necessary to confirm cross-institutional robustness.

Fourth, labels were provided at the image/ROI level rather than patient-level clinical outcomes, and the reference standard was not a vascular imaging modality. Although high AUC/AUPRC were obtained, panoramic radiography cannot replace carotid ultrasound or CT angiography, which remain the diagnostic standards for plaque characterization and stenosis quantification.

Finally, the model is a two-dimensional CNN (MobileNetV2) initialized with ImageNet weights. While computationally efficient and well-suited to limited datasets, it does not exploit 3D or temporal information and may miss context outside the ROI. Future work could evaluate alternative backbones, self-supervised pretraining on dental radiographs, test-time adaptation, and end-to-end pipelines operating on full panoramic images, alongside prospective, multicenter validation and health-economic analysis of different decision thresholds.

### 4.5. Future Directions

Although the present study demonstrates the technical feasibility of deep learning-based opportunistic screening for carotid atheroma on panoramic radiographs, further steps are required before clinical deployment. Future work should first prioritize dataset expansion via multicenter cohorts, capturing broader variation in acquisition protocols, vendors, and patient demographics. Such diversity is essential to stress-test robustness and reduce domain shift, enabling external validation and site-specific threshold calibration for clinically meaningful operating points. As an ethical alternative to centralized data sharing, federated learning—with secure aggregation and optional differential-privacy noise—can train shared models across institutions while keeping patient data on-premises, thereby facilitating multicenter collaboration without transferring protected health information.

Second, integrating explainable AI (XAI)—e.g., Grad-CAM/Score-CAM saliency, occlusion sensitivity—can localize cues within the C3–C4 ROI and support clinician trust, error analysis, and iterative annotation refinement. Coupling XAI with uncertainty estimation (Monte Carlo dropout, deep ensembles) would allow abstention or referral when predictions are low-confidence.

Third, moving beyond single-modality inputs, multimodal models that fuse panoramic ROI cues with clinical variables (age, sex, vascular risk factors) or complementary imaging (periapical views, CBCT where available) may improve discrimination and calibration. In parallel, self-supervised pretraining on large unlabeled dental radiograph corpora could yield stronger feature extractors than generic ImageNet initialization on grayscale ROIs.

Fourth, the current ROI-based approach could be extended to full-panoramic pipelines (automatic ROI proposal + classification), accompanied by image-quality control (artifact/marker detection) and test-time adaptation to mitigate site-specific shifts. Active learning strategies may also reduce the labeling burden by prioritizing the most informative cases for expert review.

Finally, prospective implementation studies are needed to evaluate real-world performance, workflow integration (e.g., PACS/RIS or dental imaging software), and health–economic impact across operating points (Youden vs. high-sensitivity). Reporting should follow emerging AI in medicine guidelines, for instance, through transparent reporting of a multivariable prediction model for individual prognosis or diagnosis–artificial intelligence (TRIPOD-AI)/consolidated standards of reporting trials–artificial intelligence (CONSORT-AI), with attention to fairness audits across subgroups and clear data-governance practices to support safe, reproducible deployment.

## 5. Conclusions

This study has developed and validated a MobileNetV2-based convolutional neural network for automated detection of carotid atheromas in panoramic dental radiographs using a two-stage fine-tuning schedule on ROI crops (640 × 320). Despite the limited and heterogeneous dataset, the model achieved strong and well-balanced performance on the independent test set (AUC ≈ 0.96–0.97, AUPRC ≈ 0.97), supporting its suitability for opportunistic screening in dental workflows.

Methodologically, the work demonstrates that a lightweight CNN with ImageNet initialization, conservative preprocessing, class-weighted training, and optional test-time augmentation can yield robust performance under the real-world constraints of small, variable datasets. Clinically, the findings reinforce the feasibility of leveraging routine panoramic radiographs as a low-cost triage channel for vascular risk, enabling timely referral from dental settings and strengthening the interface between oral and general health.

Interpretation should remain cautious given the sample size, single-framework training/evaluation, and lack of external validation. Future research should emphasize multicenter and prospective validation, explainability, uncertainty quantification, and workflow integration studies. Ultimately, this work supports the vision of integrating dental imaging into broader preventive health frameworks, bridging oral and systemic care through explainable AI.

## Figures and Tables

**Table 1:** Training/validation performance at representative epochs (primary metric = val AUC).

Phase	Epoch	Train *ACC*	Val *ACC*	Val AUC	Train Loss	Val Loss
1—Frozen	1	0.58	0.53	0.58	0.77	0.70
	6	0.63	0.74	0.77	0.67	0.63
	13	0.67	0.60	0.81	0.62	0.68
	19	0.72	0.60	0.84	0.57	0.70
2—Fine-tuning	1	0.75	0.47	0.84	0.51	0.79
	3	0.81	0.61	0.85	0.49	0.71
	10	0.80	0.54	0.86	0.48	0.84
	13 *	0.97	0.70	0.95	0.14	0.73

* Best validation AUC (checkpoint used for evaluation).

**Table 2:** Classification metrics on the independent test set.

Metric	Youden J (thr ≈ 0.84)	*SEN* ≈ 0.80 (thr ≈ 0.98)
*ACC*	94.7%	91.2%
*PRE* (PPV)	91.7%	95.0%
*SEN*	95.7%	82.6%
*SPE*	94.1%	97.1%
*F*1	93.6%	88.3%
AUC	0.963	0.968
AUPRC	0.968	0.970

## Data Availability

The code used for data preprocessing, model training, inference, and Grad-CAM visualization is publicly available at the following repository: [https://github.com/thais-ufu/carotid-atheroma-screening-panoramic-xray] (accessed on 12 January 2026). The datasets analyzed in this study are publicly available on Zenodo (see [34,35]). This repository provides detailed instructions for reproducing the fixed train/validation/test split.

## Abbreviations

The following abbreviations are used in this manuscript:

ACC	Accuracy
AI	Artificial Intelligence
AUC	Area Under the Receiver Operating Characteristic Curve
AUPRC	Area Under the Precision–Recall Curve
ANOVA	Analysis of Variance
BN	Batch Normalization
CLAHE	Contrast Limited Adaptive Histogram Equalization
CNN	Convolutional Neural Networks
CONSORT-AI	Consolidated Standards of Reporting Trials–Artificial Intelligence
CPU	Central Processing Unit
CTA	Computed Tomography Angiography
FLOP	Floating point operation
FP	False Positive
FN	False Negative
GPU	Graphics Processing Unit
LVAT	Latent-Space Virtual Adversarial Training
MobileNetV2	A lightweight convolutional neural network architecture optimized for efficient image classification, developed by Google (2018).
MRA	Magnetic Resonance Angiography
NPV	Negative Predictive Value
PACS	Picture Archiving And Communication System
PPV	Positive Predictive Value
PR	Precision–Recall
RIS	Radiology Information System
ROC	Receiver Operating Characteristic
ROI	Region of Interest
SEN	Sensitivity
SPE	Specificity
TN	True Negative
TP	True Positive
TRIPOD-AI	Transparent Reporting of a Multivariable Prediction Model for Individual Prognosis or Diagnosis–Artificial Intelligence
TTA	Test Time Augmentation
XAI	Explainable Artificial Intelligence
WHO	World Health Organization
